# The combination of metabolic syndrome and inflammation increased the risk of colorectal cancer

**DOI:** 10.1007/s00011-022-01597-9

**Published:** 2022-06-18

**Authors:** Tong Liu, Yali Fan, Qingsong Zhang, Yiming Wang, Nan Yao, Mengmeng Song, Qi Zhang, Liying Cao, Chunhua Song, Hanping Shi

**Affiliations:** 1 Department of Gastrointestinal Surgery/Clinical Nutrition, Capital Medical University Affiliated Beijing Shijitan Hospital, Beijing, 100038 China; 2 Beijing International Science and Technology Cooperation Base for Cancer Metabolism and Nutrition, Beijing, 100038 China; 3 Key Laboratory of Cancer FSMP for State Market Regulation, Beijing, 100038 China; 4 Department of General Surgery, Aerospace Center Hospital, Beijing, 100038 China; 5 Department of General Surgery, Kailuan General Hospital, Tangshan, 063000 China; 6 Department of Hepatological Surgery, Kailuan General Hospital, Tangshan, 063000 China; 7 Department of Epidemiology and Statistics, College of Public Health, Zhengzhou University, Zhengzhou, 450001 Henan China

**Keywords:** Colorectal cancer, Inflammation, Metabolic syndrome, Joint-effect

## Abstract

**Background:**

Inflammation and metabolic syndrome (MetS) may act synergistically and possibly accelerate the initiation and progression of colorectal cancer (CRC). We prospectively examined the joint effect of MetS and inflammation on the risk of CRC.

**Methods:**

We studied 92,770 individuals from the Kailuan study. MetS was defined based on the presence of three or more of the following components. (1) high glucose: FPG > 5.6 mmol/L; (2) high blood pressure: SBP ≥ 130 mmHg or DBP ≥ 85 mmHg; (3) high triglycerides: triglycerides > 1.69 mmol/L; (4) low HDL-C: HDL-C < 1.04 mmol/L in men or 1.29 mmol/L in women; and (5) visceral adiposity: waist circumference ≥ 85 cm in men or 80 cm in women. Inflammation was defined as hs-CRP ≥ 3 mg/L. We divided participants into four groups for the primary exposure according to the presence/absence of inflammation and presence/absence of MetS. Cox proportional hazards regression models were used to evaluate the association of MetS and/or inflammation with the risk of CRC.

**Results:**

Compared with metabolically healthy noninflammatory individuals, inflammatory participants without MetS and inflammatory participants with MetS were associated with a 1.3-fold and 4.18-fold increased risk of CRC with corresponding HRs (95% CI) of 1.34 (1.09, 1.64) and 4.18 (3.11, 5.62), respectively. The combination of MetS and inflammation was associated with the highest risk of CRC in all subgroups, especially among participants who were female, in younger age, and obese. Sensitivity analyses further validated our primary findings.

**Conclusions:**

We found the combination of MetS and inflammation could significantly increase the risk of CRC. Including CRP in the diagnosis of MetS may help to identify additional high-risk participants who should be targeted for early diagnosis and prevention of CRC.

*Trial registration *Kailuan study, ChiCTR–TNRC–11001489. Registered 24 August, 2011-Retrospectively registered, http:// www.chictr.org.cn/showprojen.aspx?proj=8050

**Supplementary Information:**

The online version contains supplementary material available at 10.1007/s00011-022-01597-9.

## Introduction

Colorectal cancer (CRC) is the third most frequent malignancy in both men and women worldwide [1], and ranks second in terms of mortality, causing 880,000 deaths in 2018 [2]. The significant increase in morbidity from CRC in China may be due to changes in risk factors, including poor diet [3, 4] (low consumption of fruits, fiber and vegetables, and high consumption of processed meats), lack of physical activity [5], and the increasing prevalence of obesity [6]. In addition, data from epidemiological, experimental, and clinical investigations supports the concept that metabolic syndrome (MetS) plays an important role in the development and progression of CRC [7–9]; however, the existence of discordant results may suggest the existence of high-risk subgroups of individuals with MetS.

MetS is not a disease per se but is a group of metabolic risk factors defined by hypertension, central obesity, dyslipidemia, and hyperglycemia states. In addition, accumulating evidence suggests a link between MetS or its components and the development of persistent low-grade inflammation [10, 11]. C-reactive protein (CRP) is the most extensively used biomarker of inflammation [12]. Recent epidemiologic studies have found a link between circulating high-sensitivity CRP (hs-CRP), which is CRP assessed with a high-sensitivity assay, and an elevated risk of CRC [13, 14]. Inflammation and MetS may act synergistically and possibly accelerate the initiation and progression of malignancies. However, no previous study has examined the impact of MetS coupled with inflammation on the risk of CRC, which is critical because people with metabolic dysfunction and inflammation may be more likely to develop CRC.

The Kailuan study is a prospective, population-based cohort study with follow-ups conducted every 2 years. The measurements of the components of MetS and hs-CRP provide us with a valuable opportunity to examine whether the four categories defined by the presence/absence of MetS with the presence/absence of inflammation are related to the occurrence of CRC. We hypothesized that metabolically unhealthy participants with inflammation would have a higher risk of CRC.

## Methods

### Study population

The Kailuan study explored risk factors for chronic diseases including cancer. The study’s design and procedures were previously described [15]. In all, a total of 101,510 Kailuan Group employees (81,110 males and 20,400 women, ages 18 to 98) were invited to participate in the baseline health assessment between July 2006 and October 2007, and biennial follow-ups. At baseline and each follow-up, each subject was assessed using standardized questionnaires, clinical examinations, and laboratory tests.

**Fig. 1:**
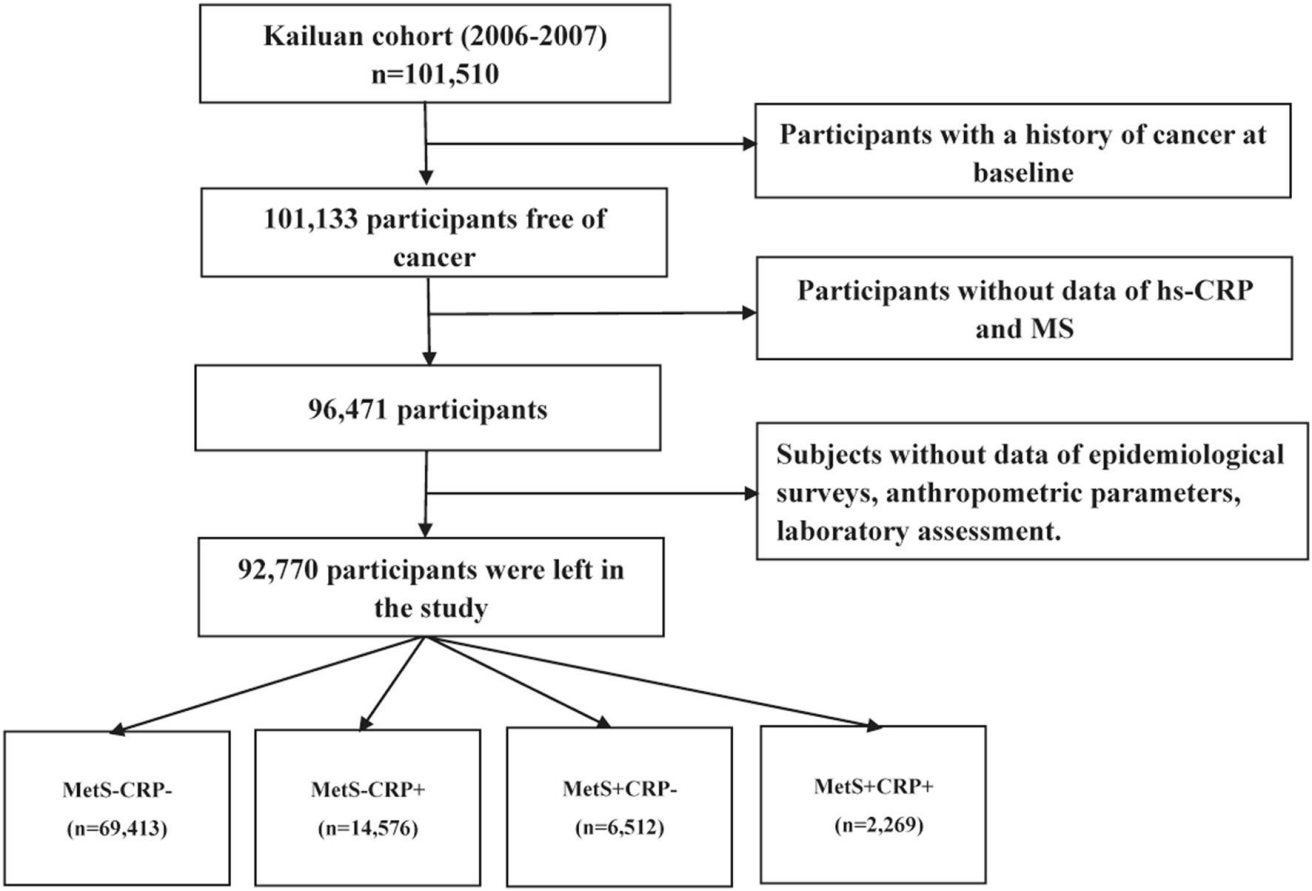
Flow chart of study participants

In this study, we excluded 377 individuals who had a history of malignancy at the time of the baseline examination. We also excluded 4,662 participants who did not have measurements of MetS components, including waist circumference (WC), systolic blood pressure (SBP), diastolic blood pressure (DBP), triglyceride (TG), fasting plasma glucose (FPG), high-density lipoprotein cholesterol (HDL-C), and hs-CRP which was used as an indicator of inflammation in this study. In addition, we excluded 6,493 participants who lacked information about other potential confounders including age, sex, BMI, total cholesterol (TC), alanine aminotransferase (ALT), uric acid (UA), family personal income, educational background, marital status, smoking status, drinking status, physical activity, sedentary lifestyle, tea consumption, high-fat diets, and family history of malignancy. Eventually, this study enrolled 92,770 individuals, including 18,638 women and 74,132 males (Fig. 1).

The ethics committees at Kailuan General Hospital and Beijing Shijitan Hospital approved the protocol for this study, which followed the principles of the Helsinki Declaration. All participants signed informed consent forms. All the authors in this current study had access to the study data and reviewed and approved the final manuscript.

### Collection and definitions of MetS and inflammation

WC was measured with a tape measure midway between the lowest rib and the pelvis. Each participant’s blood pressure was taken twice in the sitting position using a calibrated mercury sphygmomanometer on the left arm. Two consecutive blood pressure readings were collected, and the average of the two values was analyzed. Hypertension was defined as a SBP ≥ 140 mm Hg, a DBP ≥ 90 mm Hg, or a self-reported history of hypertension [16]. Blood samples were taken after fasting overnight (8–12 h) using vacuum tubes containing EDTA, separated and kept at − 80 °C for further analysis. All blood samples were analyzed using an auto-analyzer (Hitachi 747; Hitachi, Tokyo, Japan) at Kailuan General Hospital’s central laboratory. An FPG level ≥ 7.0 mmol/L, use of oral hypoglycemic medications or insulin, or a validated physician diagnosis were all used to diagnose diabetes mellitus. A high-sensitivity nephelometry test was used to measure serum hs-CRP (Cias Latex CRP-H, Kanto Chemical Co. Inc, Tokyo, Japan). According to the Centers for Disease Control and Prevention and the American Heart Association guidelines, low-grade inflammation was defined as hs-CRP ≥ 3 mg/L [17].

According to the third report of the adult education group of the American Cholesterol Education Program (NCEP-ATP III) [16], MetS was defined based on the presence of three or more of the following components: (1) high glucose: FPG > 5.6 mmol/L or diabetes diagnosis previously; (2) high blood pressure: SBP ≥ 130 mmHg or DBP ≥ 85 mmHg, or hypertension diagnosis previously; (3) high triglycerides: triglycerides > 1.69 mmol/L; (4) low HDL-C: HDL-C < 1.04 mmol/L in men or 1.29 mmol/L in women; and (5) visceral adiposity: waist circumference ≥ 85 cm in men or 80 cm in women.

We divided patients into four groups for the primary exposure according to the presence/absence of inflammation (hs-CRP > 3 mg/L) and presence/absence of MetS: (MetS-CRP-: participants without MetS and with hs-CRP ≤ 3 mg/L; MetS-CRP + : participants without MetS and with hs-CRP > 3 mg/L; MetS + CRP-: participants with MetS and with hs-CRP ≤ 3 mg/L; MetS + CRP + : participants with MetS and with hs-CRP > 3 mg/L). We totaled the MetS components from 0 (no abnormality) to 5 (all abnormalities) to assess the dose–response relationship of the degree of metabolic dysregulation with the risk of CRC. Due to few participants having scores of 4 or 5, these participants were grouped together.

### Outcome ascertainment

The following methods were used to identify incident CRC cases: (1) checking clinical examination participants took every 2 years until December 31, 2019; (2) checking medical records from the Tangshan medical insurance system and the Kailuan Social Security Information System yearly; and (3) reviewing death certificates from the Provincial Vital Statistics Offices (PVSO) once a year to obtain additional missing information. According to the International Classification of Diseases, Tenth Revision (ICD-10), clinical experts assessed the diagnosis and categorized CRC patients as C18–21.

### Potential confounders

A standard questionnaire was used to collect information on each participant's age, sex, socioeconomic situation, educational background, living habits, and personal and family members’ medical histories. Drinking and smoking status was divided into four groups: never, past, moderate, and severe (1 time/day or 1 cigarette/day). Physical activity was classified as never, occasionally, or regularly (≥ 3 times/week, ≥ 30 min/time). A sedentary lifestyle was divided into three categories: < 4 h/day, 4–8 h/day, and > 8 h/day. Tea consumption was divided into four categories: never, < 1 time/month, 1–3 times/month, 1–3 times/week, and > 4 times/week. High-fat diets were classified into three groups: seldom, occasionally, and regularly based on the response towards the question of frequency of high-fat diets.

Qualified medical personnel assessed the height and weight of all participants using conventional procedures. BMI was measured as the ratio of body weight (kg) to the square of height (m^2^) and was separated into three categories: normal weight (< 24 kg/m^2^), overweight (24.00–27.99 kg/m^2^), and obese (28 kg/m^2^).

The concentration of TC was measured by the colorimetric enzymatic method (Mind Bioengineering Co. Ltd, Shanghai, China). ALT (ALT, in U/L) was measured with an enzymatic rate method. The UA concentrations were measured with the oxidase method. The tertiles of each variable were used to separate the serum TC, ALT, and UA levels into three groups.

### Statistical analysis

The mean ± standard deviation was used to represent normally distributed variables and one-way analysis of variance (ANOVA) was used to compare the difference among groups. Median (interquartile range) was used to describe the skewed distribution variables (hs-CRP and TG) and were compared using nonparametric tests. Absolute values with percentages were used to describe categorical variables and compared using the Chi-square test. Person-years were computed from the date of baseline examination through the date of CRC diagnosis, death, or the 31st of December 2019, whichever event occurred first. Cox proportional hazards models were used to estimate the hazard ratios (HRs) and their 95% confidence intervals (CIs) for CRC. Subgroup analyses were conducted by stratifying participants by sex (man vs. women), and age (≤ 45 years, 45–65 years, and > 65 years).

In the sensitivity analysis, we excluded participants who had cancer within the first year of follow-up to eliminate the possibility of reverse causation. We also excluded participants who took statins, or received antihypertensive medications, oral hypoglycemic agents, or insulin to eliminate the possible effect of medication on the levels of MetS components.

A *P*-value (two-sided) < 0.05 was considered statistically significant. Statistical analyses were performed using a commercially available software program (SAS software, version 9.4).

## Results

A total of 92,770 individuals were separated into four groups: MetS-CRP- (*n* = 69,413), MetS-CRP + (*n* = 14,576), MetS + CRP- (*n* = 6,512), and MetS + CRP + (*n* = 2,269). The baseline characteristics of the participants are summarized in Table 1. The study population’s average age was 51.48 ± 12.44 years. Significant differences were found in age, sex, and levels of hs-CRP, WC, FBG, SBP, DBP, HDL-C, TG, TC, ALT, UA, and BMI. In addition, the percentages of educational background, marital status, reported income, physical activity, sedentary lifestyle, tobacco consumption, alcohol consumption, tea consumption, high-fat diets, salt intake, hypertension, and diabetes mellitus differed significantly across four the prespecified groups. However, no difference in the prevalence of family history of cancer was observed among the four groups.

**Table 1:** Baseline characteristics of the participants stratified by MetS and hs-CRP status

Variables	MetS-CRP−	MetS-CRP +	MetS + CRP−	MetS + CRP +	*P*-value
n (%)	69,413	14,576	6,512	2,269	
Age (year)	50.50 ± 12.46	54.77 ± 13.04	53.12 ± 9.69	55.61 ± 10.00	< 0.001
Hs-CRP (mg/L)	0.55(0.22,1.13)	5.92(4.00,9.14)	0.83(0.38,1.55)	5.80(3.89,8.80)	< 0.001
WC (cm)	85.88 ± 9.67	89.26 ± 10.56	90.72 ± 8.88	93.85 ± 9.86	< 0.001
FBG (mmol/L)	5.32 ± 1.43	5.41 ± 1.76	6.84 ± 1.81	7.18 ± 1.92	< 0.001
SBP (mmHg)	128.79 ± 20.06	131.94 ± 21.44	147.49 ± 20.09	148.40 ± 21.56	< 0.001
DBP (mmHg)	82.50 ± 11.31	83.17 ± 11.73	93.28 ± 11.11	92.65 ± 11.40	< 0.001
HDL-C (mmol/L)	1.55 ± 0.39	1.55 ± 0.41	1.51 ± 0.43	1.53 ± 0.47	< 0.001
TG (mmol/L)	1.52(1.29,1.77)	1.50(1.28,1.76)	1.44(1.22,1.75)	1.46(1.23,1.76)	< 0.001
Male (%)	57,299(82.55)	12,115(83.12)	3668(56.33)	1050(46.28)	< 0.001
Reported income (¥)					< 0.001
< 600	20,607(29.69)	3850(26.41)	1828(28.07)	522(23.01)	
600–800	38,815(55.92)	8668(59.47)	3822(58.69)	1426(62.85)	
800–1000	5315(7.66)	1129(7.75)	479(7.36)	173(7.62)	
> 1000	4676(6.74)	929(6.37)	383(5.88)	148(6.52)	
Marital status (%)					< 0.001
Never	1340(1.93)	193(1.32)	17(0.26)	8(0.35)	
Married	65,568(94.46)	13,631(93.52)	6204(95.27)	2133(94.01)	
Divorced	587(0.85)	126(0.86)	53(0.81)	28(1.23)	
Widowed	1219(1.76)	445(3.05)	168(2.58)	81(3.57)	
Remarried	699(1.01)	181(1.24)	70(1.07)	19(0.84)	
Educational background (%)					< 0.001
Never	760(1.09)	286(1.96)	63(0.97)	27(1.19)	
Primary school	6282(9.05)	1768(12.13)	652(10.01)	233(10.27)	
Middle school	48,195(69.43)	9761(66.97)	4781(73.42)	1633(71.97)	
High school	9223(13.29)	1785(12.25)	772(11.86)	279(12.30)	
College graduate or above	4953(7.14)	976(6.70)	244(3.75)	97(4.28)	
TC (%)					< 0.001
< 4.51 mmol/L	23,851(34.36)	5067(34.76)	1570(24.11)	523(23.05)	
4.51 ~ 5.34 mmol/L	23,675(34.11)	4876(33.45)	1824(28.01)	664(29.26)	
> 5.34 mmol/L	21,887(31.53)	4633(31.79)	3118(47.88)	1082(47.69)	
ALT (%)					< 0.001
< 14.90 u/L	23,326(33.60)	5177(35.52)	1785(27.41)	630(27.77)	
14.90 ~ 22.00 u/L	24,644(35.50)	4811(33.01)	2229(34.23)	726(32.00)	
> 22.00 u/L	21,443(30.89)	4588(31.48)	2498(38.36)	913(40.24)	
UA (%)					< 0.001
< 249.40 μmol/L	23,227(33.46)	4700(32.24)	2207(33.89)	759(33.45)	
249.40 ~ 317.00 μmol/L	23,914(34.45)	4471(30.67)	2076(31.88)	704(31.03)	
> 317.00 μmol/L	22,272(32.09)	5405(37.08)	2229(34.23)	806(35.52)	
BMI (%)					< 0.001
< 24 kg/m^2^	29,715(42.81)	5371(36.85)	1142(17.54)	282(12.43)	
24–28 kg/m^2^	28,764(41.44)	6004(41.19)	3081(47.31)	1021(45.00)	
> 28 kg/m^2^	10,934(15.75)	3201(21.96)	2289(35.15)	966(42.57)	
Physical exercise (%)					< 0.001
Never	6345(9.14)	1117(7.66)	493(7.57)	138(6.08)	
Occasionally	52,201(75.20)	11,306(77.57)	4834(74.23)	1771(78.05)	
Regularly	10,867(15.66)	2153(14.77)	1185(18.20)	360(15.87)	
Smoking status (%)					< 0.001
Never	40,262(58.00)	8844(60.68)	4596(70.58)	1763(77.70)	
Past	3921(5.65)	956(6.56)	314(4.82)	91(4.01)	
Moderate	2628(3.79)	439(3.01)	176(2.70)	42(1.85)	
Severe	22,602(32.56)	4337(29.75)	1426(21.90)	373(16.44)	
Drinking status (%)					< 0.001
Never	39,463(56.85)	8969(61.53)	4536(69.66)	1750(77.13)	
Past	2633(3.79)	712(4.88)	187(2.87)	63(2.78)	
Moderate	14,235(20.51)	2545(17.46)	821(12.61)	217(9.56)	
Severe	13,082(18.85)	2350(16.12)	968(14.86)	239(10.53)	
Sedentary lifestyle (%)					< 0.001
< 4 h/day	51,491(74.18)	11,189(76.76)	4931(75.72)	1756(77.39)	
4–8 h/day	15,625(22.51)	2960(20.31)	1386(21.28)	427(18.82)	
> 8 h/day	2297(3.31)	427(2.93)	195(2.99)	86(3.79)	
Tea consumption (%)					< 0.001
Never	51,709(74.49)	11,045(75.78)	5116(78.56)	1819(80.17)	
< 1 time/month	3266(4.71)	606(4.16)	212(3.26)	74(3.26)	
1–3 times/month	4271(6.15)	905(6.21)	330(5.07)	98(4.32)	
1–3 times/week	3555(5.12)	659(4.52)	288(4.42)	80(3.53)	
> 4 times/week	6612(9.53)	1361(9.34)	566(8.69)	198(8.73)	
High-fat diets (%)					< 0.001
Seldom	5954(8.58)	1133(7.77)	564(8.66)	193(8.51)	
Occasionally	56,920(82.00)	12,201(83.71)	5315(81.62)	1908(84.09)	
Regularly	6539(9.42)	1242(8.52)	2326(6.79)	168(7.40)	
Salt intake (%)					< 0.001
Low (< 6 g/day)	6539(9.42)	1220(8.38)	605(9.29)	189(8.34)	
Intermediate (6–10 g/day)	55,250(79.63)	11,828(81.23)	5226(80.29)	1857(81.95)	
High (> 10 g/day)	7624(10.98)	1528(10.48)	681(10.46)	223(9.83)	
Family history of cancer (%)	2531(3.65)	545(3.74)	277(3.49)	85(3.75)	0.828
Diabetes mellitus (%)	3986(5.74)	1189(8.16)	1785(27.41)	765(33.72)	< 0.001
Hypertension (%)	24,932(35.92)	6213(42.62)	5311(81.56)	1840(81.09)	< 0.001

*Hs-CRP* high-sensitivity C-reactive protein, *W*C waist circumference, *FBG* fasting blood glucose, *SBP* systolic blood pressure, *DBP* diastolic blood pressure, *HDL-C* high-density lipoprotein cholesterol, *TG* triglyceride, *BMI*: body mass index, *TC*, total cholesterol, *ALT* alanine aminotransferase, *SUA* serum uric acid

The median (IQR) duration of follow-up was 13.02 (12.70, 13.20) years. At the end of the study, 626 new-onset CRC cases were identified. The crude and adjusted HRs (95% CI) for the association between MetS components, MetS, or hs-CRP and the risk of CRC are shown in Table 2. Compared with participants who had 0 metabolic risk factor, individuals who had 3 and 4 (5) metabolic risk factors were associated with a 2.0-fold (HR = 2.02, 95% CI 1.52–2.69) and 2.7-fold (HR = 2.72, 95% CI 1.60–4.64) elevated risk of CRC in the multivariate analyses. Compared with metabolically healthy participants (without MetS), the adjusted HR (95% CI) for the association of MetS with CRC risk was 1.86 (1.49 ~ 2.34) even after adjusting for hs-CRP and other confounders. A significant interaction between MetS and inflammation (hs-CRP > 3 mg/L) was found for the risk of CRC (*P* for interaction < 0.001). In addition, a significant association was observed of hs-CRP (per SD increment) and elevated hs-CRP (> 3 mg/L) with the risk of incident CRC in the multivariate analyses even though adjustments were made for MetS. Table S1 shows the association of each MetS component with the development of CRC. In the adjusted models, abdominal obesity, high glucose, and low HDL-C were associated with the risk of incident CRC. However, only abdominal obesity and low HDL-C remained statistically significant in the mutual adjustment model that incorporates all metabolic risk factors.

**Table 2:** Hazard ratios (HRs) for the association of MetS or its components or hs-CRP levels with CRC risk

Group	Cases/person-years	Crude models		Adjusted models	
		HR (95% CI)	*p*-value	HR (95% CI)	*p*-value
MetS metrics^a^					
MetS-0	152/349088	Ref		Ref	
MetS-1	219/418573	1.21(0.98,1.48)	0.077	1.13(0.92,1.39)	0.257
MetS-2	149/261098	**1.32(1.05,1.65)**	0.017	1.20(0.94,1.51)	0.138
MetS-3	87/91177	**2.21(1.70,2.87)**	< 0.001	**2.02(1.52,2.69)**	< 0.001
MetS-4 (5)	19/15120	**2.89(1.79,4.65)**	< 0.001	**2.72(1.60,4.64)**	< 0.001
* P* for trend			< 0.001		< 0.001
MetS^a^					
0	520/1028759	Ref		Ref	
1	106/106297	**1.98(1.61,2.44)**	< 0.001	**1.86(1.49,2.34)**	< 0.001
*P* for interaction^b^					< 0.001
Hs-CRP^c^					
≤ 3 mg/L	442/936212	Ref		Ref	
> 3 mg/L	184/198844	**2.00(1.68,2.37)**	< 0.001	**1.62(1.36,1.93)**	< 0.001
Hs-CRP (per SD)	626/1135056	**1.06(1.03,1.09)**	< 0.001	**1.05(1.01,1.08)**	0.011

Adjustments were a made for age (every 10 years), sex, family income, educational background, marital status, BMI, TC, ALT, SUA, smoking status, drinking status, physical activity, sedentary lifestyle, tea consumption, salt intake, high-fat diet, family history of cancer in the adjusted models

^a^Further adjusted for hs-CRP (≤ 3 vs. > 3)

^b^Interaction between MetS and hs-CRP

^c^Further adjusted for MetS

Table 3 shows the crude and multivariable-adjusted associations of the primary exposure with CRC risk. Compared with metabolically healthy noninflammatory individuals, inflammatory participants without MetS, and inflammatory participants with MetS were associated with a 1.3-fold and 4.18-fold increased risk of CRC with the corresponding HRs (95% CI) of 1.34 (1.09, 1.64) and 4.18 (3.11, 5.62), respectively.

**Table 3:** Hazard ratios (HRs) for the association of MetS and inflammation with CRC risk

Group	Cases/person-years	Crude models		Adjusted models	
		HR (95% CI)	*p*-value	HR (95% CI)	*p*-value
MetS(−) CRP(−)	395/856820	Ref		Ref	
MetS(−) CRP(+)	125/171939	**1.61(1.31,1.96)**	< 0.001	**1.34(1.09,1.64)**	< 0.001
MetS(+) CRP(−)	47/79392	1.29(0.95,1.74)	0.101	1.24(0.91,1.69)	0.179
MetS(+) CRP(+)	59/26905	**4.84(3.68,6.36)**	< 0.001	**4.18(3.11,5.62)**	< 0.001

Results presented with bold valued were statistically significant with all *p* value < 0.05

Adjustments were made for age (every 10 years), sex, family income, educational background, marital status, BMI, TC, ALT, SUA, smoking status, drinking status, physical activity, sedentary lifestyle, tea consumption, salt intake, high-fat diet, family history of cancer in the adjusted models

**Fig. 2:**
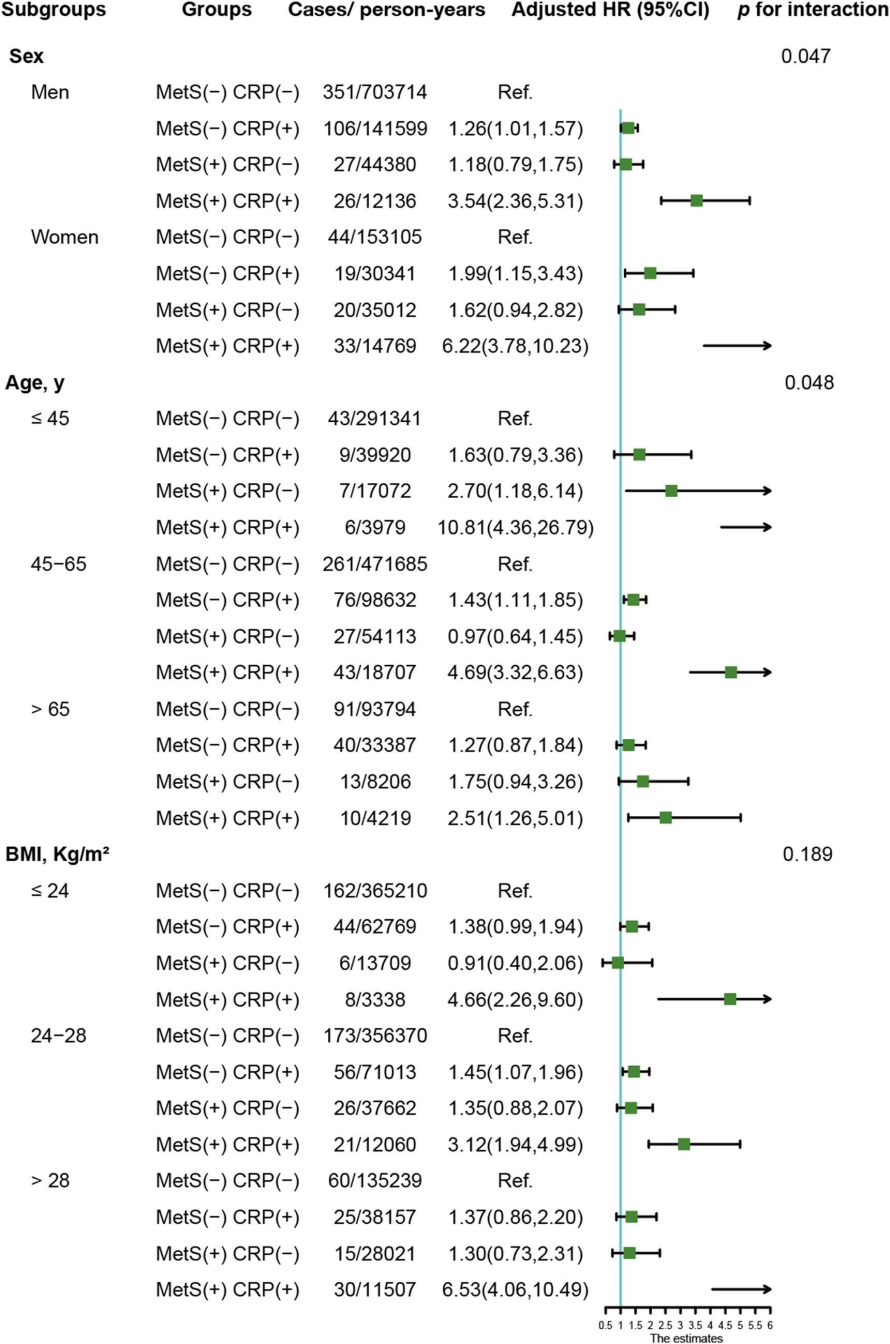
Subgroup analysis of the association of MetS and hs-CRP levels with CRC risk

**Fig. 3:**
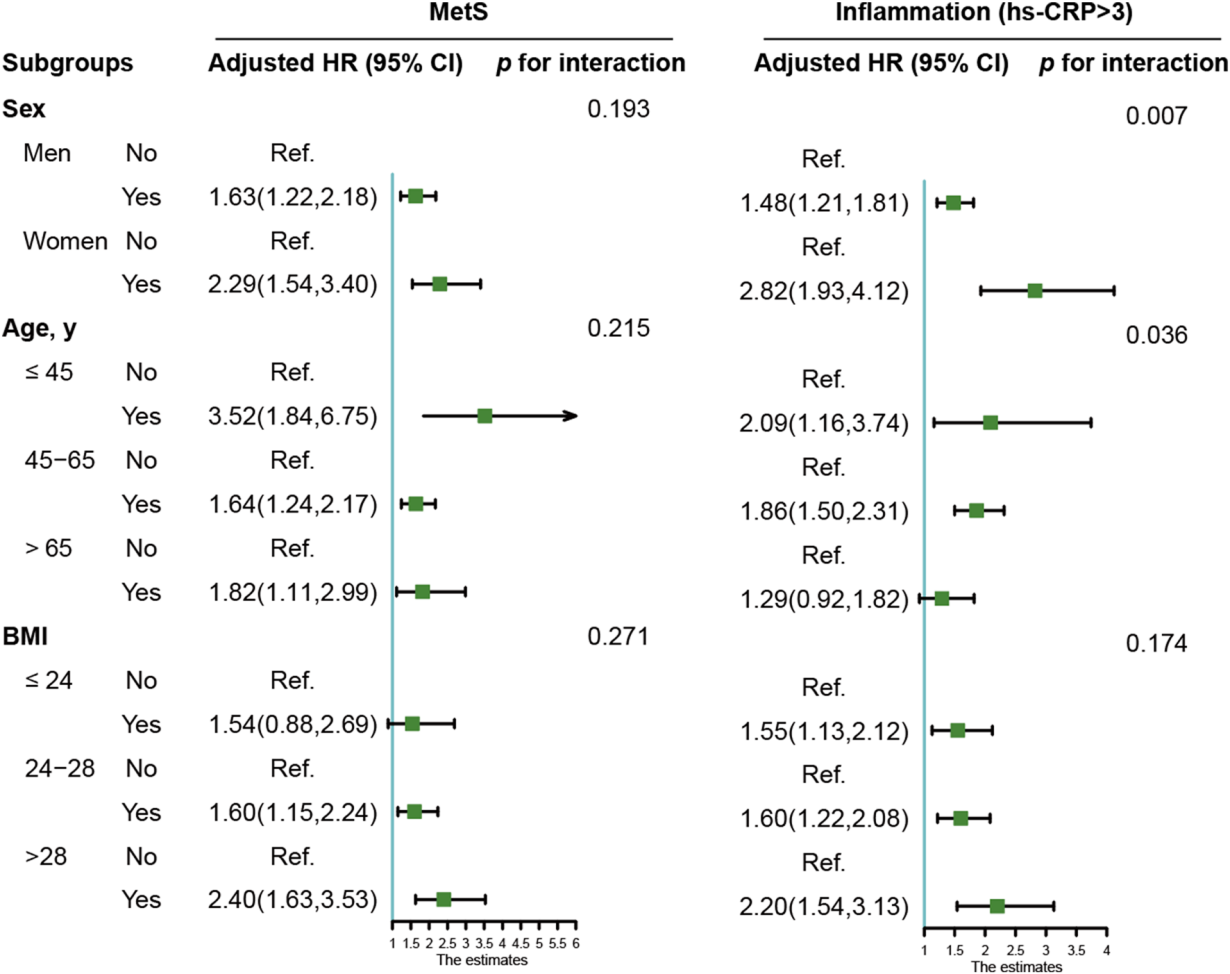
Subgroup analysis of the association of MetS or hs-CRP levels with CRC risk

Similar results were also obtained when participants were stratified by sex, age or BMI (Fig. 2). Compared with metabolically healthy non-inflammation participants, the combination of MetS and inflammation was associated with the highest risk of CRC in all subgroups, especially among participants who were female (*HR* = 6.22, 95% CI 3.78–10.23), in younger age (*HR* = 10.81, 95% CI 4.36–26.79), and obese (*HR* = 6.53, 95% CI 4.06–10.49). Figure 3 shows the subgroups analyses of the association between MetS or inflammation and CRC risk. Except for people with a normal BMI, there was a significant association between MetS and CRC risk in all subgroups. Only the older subjects showed a null connection between elevated CRP and the risk of CRC.

Sensitivity analyses did not substantially alter the conclusions and even strengthened the HR from 1.34 to a higher level (1.38–1.43) among inflammatory participants without MetS and from 4.18 to a range of 4.42–4.61 among metabolically unhealthy participants with inflammation after excluding 26 CRC cases that had occurred within the first year of follow-up, or participants who took statins (*n *= 906), or antihypertensive medications (*n *= 7,863), or hypoglycemic drugs (*n* = 2,345) (Table S3).

## Discussion

To our knowledge, this study is the first prospective cohort study to investigate the association of MetS and inflammation with CRC incidence in the Chinese population. The primary finding of this study is that inflammation (hs-CRP > 3 mg/L) and MetS act synergistically and increase the risk of CRC. In addition, the significant interaction between MetS and inflammation (hs-CRP > 3 mg/L) for the risk of CRC along with the aforementioned results indicates that inflammation may play an important role in the occurrence of CRC caused by MetS.

We found MetS increased the risk of CRC in the general population and all subgroups except the normal weight group which was consistent with previous research. MetS was shown to be associated with an elevated risk of CRC incidence in both men and women in a systematic review and meta-analysis. The risk of CRC estimates for any single factor of the syndrome was significant for higher values of obesity, glucose, and blood pressure [18]. In another meta-analysis involving 18 studies for CRC incidence conducted by Fei Han et al., MetS increased the risk of CRC incidence in male patients and female patients. For the MetS components, only obesity and hyperglycemia were associated with an elevated risk of CRC incidence in both sexes [19]. However, not all studies found that MetS increased CRC risk. By analyzing data from 27,724 participants from the Japan Public Health Center-based Prospective Study, Inoue et al. found no association between MetS and colon cancer, nor rectal cancer. Ahmed et al. failed to find a positive association between MetS and CRC risk among women in a multicenter prospective cohort study [20].

The results regarding the association of CRP with CRC were consistent with prior research. A nested case–control study conducted in Japan found that the highest quartile group of C-reactive protein was significantly associated with a subsequent risk of colon cancer compared with the lowest group [21]. By analyzing 172 CRC patients and 342 controls, a prospective nested case–control study found that the risk of colon cancer was higher in persons in the highest vs lowest quartile of CRP [14]. In contrast, Zhang et al. found a null association between increased CRP levels and subsequent CRC risk in a prospective cohort analysis of 169 colorectal cancer cases [22], as did another prospective cohort study of 189 CRC cases [23].

MetS combined with inflammation has a greater impact on the risk of CRC incidence in women (vs. men) and youth (vs. middle-aged and elderly participants). In addition, MetS or increased CRP on its own has a greater unfavorable influence on the incidence of CRC in women and young individuals. CRP levels and numbers of MetS components increased as people got older, and women exhibited relatively higher CRP levels than men (Fig. S1), which may help explain the elevated risk of CRP or MetS for developing CRC in female group. However, we do not know why young people are more susceptible to metabolic syndrome. Future experimental studies were required to investigate this phenomenon.

The underlying mechanism by which MetS combined with inflammation increases subsequent CRC risk remains unknown. A previous study demonstrated that incorporating CRP into the definition of MetS may help identify additional high-risk individuals to target preventive methods [24]. The mechanism may include MetS and inflammation. MetS might serve as a proxy marker for additional cancer risk factors such as sedentary lifestyle, consumption of high-calorie dense meals, high-fat intake, low fiber intake, and exposure to oxidative stress [25]. Obesity, particularly visceral obesity, causes persistent systemic low-grade inflammation, which is linked to the generation of inflammatory cytokines by both adipocytes and infiltrating immune cells, resulting in a carcinogenic milieu [26]. Insulin resistance, a key component of MetS, may be produced by a shift in the balance of proinflammatory and anti-inflammatory cytokines generated by central obesity. Elevated insulin levels cause a reduction in IGF-blinding proteins 1 and 2, enhancing IGF bioavailability. The IGF-1 axis has been linked to the development of several cancer types [27]. Long-term low-grade inflammation can promote tumor formation and progression by causing protein and DNA damage. Due to inflammatory mediators such as cytokines, free radicals, prostaglandins, and growth factors, critical pathways that maintain normal cellular homeostasis can be changed by genetic and epigenetic differences. These alterations include point mutations in tumor suppressor genes, DNA methylation, and post-translational changes, all of which can lead to the presence and progression of cancer [28].

This study’s primary strength is that it provides a unique perspective on the possible link of MetS and inflammation with future CRC risk. In addition, this study examines a wide variety of potential confounding variables, such as lifestyle habits and a history of cancer-related illnesses. Cancer cases were acquired via inspections of the Tangshan Medical Insurance System and the Kailuan Social Security System, which documented all relevant health information of members. Using this method, the follow-up rate was nearly 100% in this study. In addition, strengths of this study include the prospective study design, large sample size, and long-term follow-up.

Limitations of this study should also be noted. First, colon cancer and rectal cancer could not be studied individually due to a lack of data. MetS may have different carcinogenic effects on the occurrence of colon and rectal cancers. Second, other cancer-related causative variables, such as cereal, vegetable, and high-fiber foods, are not widely discussed in the Kailuan study; thus, we cannot examine confounding variables more accurately due to the absence of information on how these items are consumed. However, dietary components are strongly related to BMI, TC and TG [29]. Since those variables were adjusted in the multivariate analyses, it is possible that they had only a minor impact on the outcomes. Third, the participants were all from the Kailuan community and did not represent the Chinese population as a whole. Thus, extrapolated findings may not accurately describe the wider Chinese population.

## Conclusion

The results of this prospective cohort study showed that the combination of MetS and inflammation could significantly increase the risk of CRC. Including CRP in the diagnosis of MetS may help identify additional high-risk participants who should be targeted for early diagnosis and prevention of CRC.

## Supplementary Information

Below is the link to the electronic supplementary material.

Supplementary file1 (DOCX 18 KB)

Supplementary file2 (TIF 3831 KB)

## Data Availability

Data will be made available upon reasonable request.
