# Supplementary material for: The combination of metabolic syndrome and inflammation increased the risk of colorectal cancer
Source: Inflamm Res. 2022 Jun 18;71(7-8):899–909. doi: 10.1007/s00011-022-01597-9 (PMC9307555; doi:10.1007/s00011-022-01597-9)
Supplement: Supplementary file 1 — Supplementary file1 (DOCX 18 KB) [file 11_2022_1597_MOESM1_ESM.docx]

**Supplementary tables**

**Table S1. The association between MetS components and CRC risk**

|  | **Crude** | | |  | **Adjusted** | |  | **Mutually adjusted** | |
| --- | --- | --- | --- | --- | --- | --- | --- | --- | --- |
|  | **HR (95%CI)** | ***p*-value** | |  | **HR (95%CI)** | ***p*-value** |  | **HR (95%CI)** | ***p*-value** |
| **Visceral adiposity** | 1.22(0.98,1.52) | | 0.077 |  | **1.74(1.07,2.83)** | 0.025 |  | 1.61(0.99,2.61) | 0.056 |
| **High blood pressure** | **1.36(1.16,1.59)** | | <0.001 |  | 1.14(0.97,1.35) | 0.110 |  | 1.09(0.93,1.29) | 0.286 |
| **High glucose** | **1.58(1.35,1.86)** | | <0.001 |  | **1.42(1.21,1.68)** | <0.001 |  | **1.39(1.18,1.63)** | <0.001 |
| **High triglycerides** | **1.19(1.01,1.40)** | | 0.035 |  | 1.13(0.96,1.34) | 0.149 |  | 1.07(0.91,2.43) | 0.423 |
| **Low HDL-C** | **1.26(0.90,1.76)** | | 0.181 |  | **1.65(1.11,2.44)** | 0.013 |  | **1.64(1.11,2.43)** | 0.014 |

**Note: Adjustments were a made for age (every 10 years), sex, family income, educational background, marital status, BMI, TC, ALT, SUA, smoking status, drinking status, physical activity, sedentary lifestyle, tea consumption, salt intake, high-fat diet, family history of cancer in the adjusted models.**

**Mutually adjusted models included all MS metrics at the same time.**

**Table S2. Sensitivity analyses of the association of MetS and inflammation with CRC risk.**

|  | **Cases/person-years** | **Adjusted models** |  |
| --- | --- | --- | --- |
|  |  | **HR (95%CI)** | ***p*-value** |
| **Exclude CRC occurred within 1 year** | | | |
| **MetS(-) CRP(-)** | 376/856808 | Ref. |  |
| **MetS(-) CRP(+)** | 120/171937 | **1.41(1.15,1.74)** | 0.001 |
| **MetS(+) CRP(-)** | 47/79392 | 1.31(0.96,1.80) | 0.088 |
| **MetS(+) CRP(+)** | 57/26904 | **4.42(3.28,5.97)** | <0.001 |
| **Exclude participants who took statins** |  |  |  |
| **MetS(-) CRP(-)** | 389/850286 | Ref. |  |
| **MetS(-) CRP(+)** | 124/170106 | **1.41(1.15,1.73)** | <0.001 |
| **MetS(+) CRP(-)** | 46/78069 | 1.23(0.89,1.68) | 0.206 |
| **MetS(+) CRP(+)** | 59/26081 | **4.45(3.31,5.98)** | <0.001 |
| **Exclude participants who** **received** **antihypertensive medications** | | | |
| **MetS(-) CRP(-)** | 343/803115 | Ref. |  |
| **MetS(-) CRP(+)** | 107/155080 | **1.43(1.14,1.78)** | 0.002 |
| **MetS(+) CRP(-)** | 31/65042 | 1.19(0.81,1.73) | 0.372 |
| **MetS(+) CRP(+)** | 41/21145 | **4.61(3.27,6.50)** | <0.001 |
| **Exclude participants who received** **oral hypoglycemic agents or insulin** | | | |
| **MetS(-) CRP(-)** | 384/842306 | Ref. |  |
| **MetS(-) CRP(+)** | 119/167789 | **1.38(1.13,1.70)** | 0.002 |
| **MetS(+) CRP(-)** | 42/73933 | 1.19(0.86,1.65) | 0.306 |
| **MetS(+) CRP(+)** | 53/24777 | **4.23(3.11,5.76)** | <0.001 |

**Note: Adjustments were a made for age (every 10 years), sex, family income, educational background, marital status, BMI, TC, ALT, SUA, smoking status, drinking status, physical activity, sedentary lifestyle, tea consumption, salt intake, high-fat diet, family history of cancer in the adjusted models.**
