# Supplementary figures and images for: The combination of metabolic syndrome and inflammation increased the risk of colorectal cancer
Source: Inflamm Res. 2022 Jun 18;71(7-8):899–909. doi: 10.1007/s00011-022-01597-9 (PMC9307555; doi:10.1007/s00011-022-01597-9)

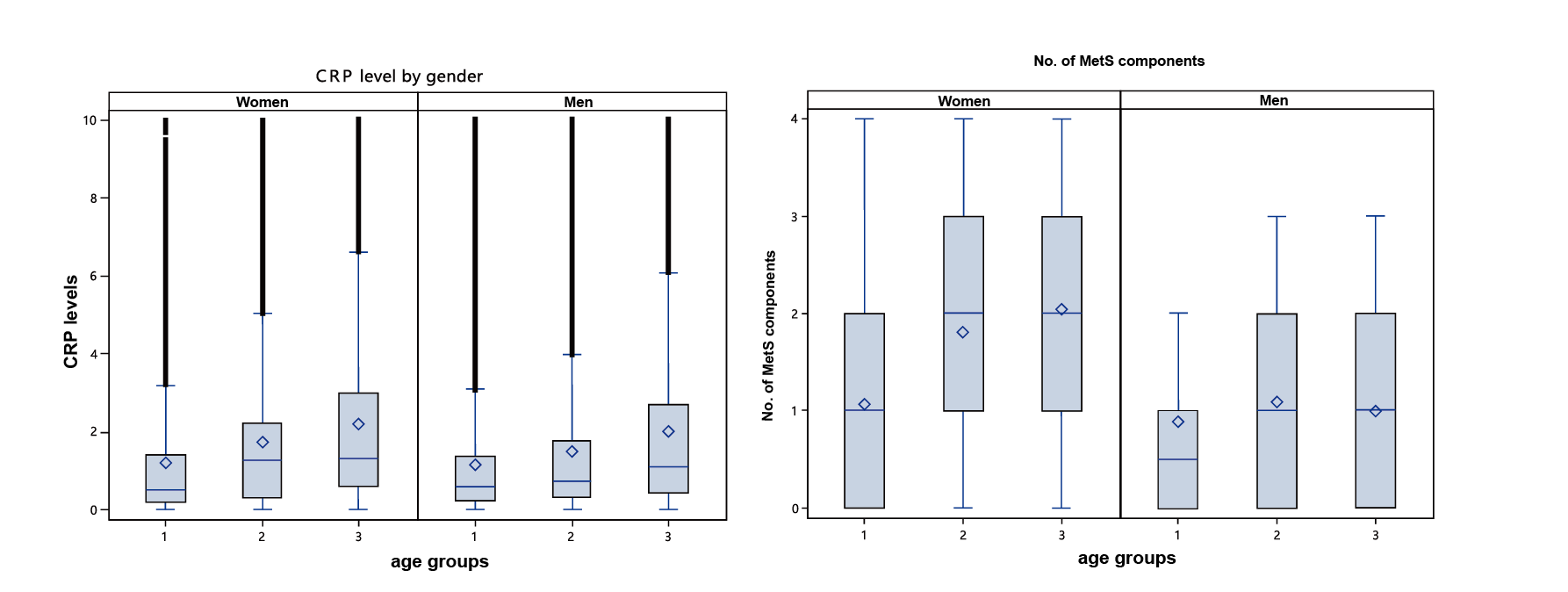

Supplement: Supplementary file 2 — Supplementary file2 (TIF 3831 KB) [file 11_2022_1597_MOESM2_ESM.tif]
